# Survey of postoperative pain control in different types of hospitals: a multicenter observational study

**DOI:** 10.1186/s12871-018-0551-3

**Published:** 2018-07-18

**Authors:** Michał Borys, Klaudia Zyzak, Agata Hanych, Michał Domagała, Piotr Gałkin, Katarzyna Gałaszkiewicz, Agata Kłaput, Kai Wróblewski, Justyna Miękina, Dariusz Onichimowski, Mirosław Czuczwar

**Affiliations:** 1 The Second Department of Anaesthesiology and Intensive Therapy, Medical University of Lublin, ul. Staszica 16, 20-081 Lublin, Poland; 2 The Department of Anaesthesia and Intensive Therapy, The Podkarpackie Center of Lung Disease, Rzeszów, Poland; 3 The Department of Anaesthesia and Intensive Therapy, Saint Lukash Hospital, Końskie, Poland; 4 The Department of Anaesthesia and Intensive Therapy, Jedrzej Sniadecki Hospital, Białystok, Poland; 5 The Department of Anaesthesia and Intensive Therapy, University of Warmia and Mazury, Olsztyn, Poland

**Keywords:** Postoperative pain management, Analgesia, Analgesics, Multimodal analgesia

## Abstract

**Background:**

Current pain assessment and treatment does not address every patient’s requirements. Although the Polish national guidelines for post-operative pain management have been published, many patients experience severe pain in the postoperative period. The main goal of our study was to assess pain severity among patients from different types of hospitals (primary, secondary, and tertiary centers) after similar types of surgeries. We also aimed to determine if there were any differences in pain severity associated with anesthesia technique, type of surgery, and the patient’s age and sex.

**Methods:**

This was a prospective, observational study. A questionnaire form was used to collect demographic data, type of hospital, surgery, anesthesia, and patient satisfaction of pain control in the postoperative period. The visual analogue scale (VAS) was used to measure pain severity at four time points after surgery (4, 8, 12, and 24 h).

**Results:**

The study was conducted from November 2015 to June 2016 in seven hospitals in Eastern Poland, and 269 women and 293 men participated. At the 4-h measurement, 39.32% of patients assessed the pain as moderate and 19.75% as severe. A difference was found in pain intensity between patients treated in primary and secondary hospitals. Vascular surgery patients had the lowest pain intensity (19 (13–26)), especially in comparison to those undergoing thoracic surgery (30 (27–33)). A sudden elevation in pain severity among patients anesthetized with single-shot spinal technique was observed. Only 4.9% of participants received strong opioids during the first 24 h after surgery.

**Conclusions:**

Postoperative pain control seems to be unexpectedly poor after single-shot subarachnoid anesthesia. Despite concerns, the use of analgesics may be insufficient in some groups of patients. Our study indicates new variables that influence the severity of pain, such as operated region, anesthetic technique, and type of surgical department. The results obtained in our study are in discrepancy with recommendations presented by the national guidelines for post-operative pain management.

**Electronic supplementary material:**

The online version of this article (10.1186/s12871-018-0551-3) contains supplementary material, which is available to authorized users.

## Background

Over 200 million major surgeries are performed worldwide annually [1]. Even though the World Health Organization proclaimed pain relief and management as a fundamental human right, many patients experience moderate or severe pain in the postoperative period [2]. According to different variables, such as type of surgery, pain-measurement tool, time of assessment, and patient’s sex and age, pain may be classified as moderate and severe in approximately 60 to 80% of cases [3–6].

Surprisingly, the introduction of new analgesic techniques and drugs, as well as the implementation of national guidelines for acute pain management, did not significantly influence inadequately-treated postoperative pain. The guidelines endorsed by the Polish Society of Anesthesiology and Intensive Therapy in 2014 comprise many analgesic approaches that are relevant in most clinical scenarios [7]. However, most data included in these guidelines were not acquired in the population of patients treated in Polish hospitals. The data presented in the recent paper by Tomaszek and Debska indicated that postoperative pain treatment is still unsatisfactory in Polish hospitals [8]. A similar conclusion was found in the study by Mędrzycka-Dabrowska and co-workers [9], which emphasizes the lack of a separate team for acute pain management and administration of insufficient quantities of medication for pain alleviation. Although pain monitoring is mandatory during the first postoperative day in all hospitals in Poland, there is no registry like that of other countries, such as Germany [10, 11]. Furthermore, it is likely that nurses, who are obliged to monitor the NRS (numerical rating scale) in the postoperative period across all surgical wards in Poland, do not have a significant impact on the prescription of analgesics or implementation of more sophisticated methods of pain control [8, 9]. According to recent papers, there are several barriers to postoperative pain management mentioned by Polish nurses [8, 9]. However, the most important factors are, in their opinion, their lack of independence in decisions concerning pharmacological treatment, as well as poor cooperation from physicians.

The first step to assess the current pain control policies in the Polish healthcare system is to acquire data from a variety of hospitals across the country. Therefore, the main goal of our study was to assess the current postoperative pain management practices after publication of the guidelines endorsed by the Polish Society of Anesthesiology and Intensive Therapy in 2014.

## Materials and methods

### Study design

Ethical approval for this study (permit number KE-0254/282/2015) was provided by the Medical University of Lublin Ethics Committee. This was a prospective, observational, multi-center study, that involved postoperative patients who underwent scheduled surgeries in seven hospitals across eastern Poland. The data were collected by medical students, after obtaining written consent from eligible patients.

### Patients and data collection

The questionnaire form contained seven sections as follows: (1) patient demographics; (2) type of anesthesia; (3) type of operated region; (4) pain evaluation with visual-analogue scale (VAS); (5) drugs, routes of their administration, and techniques used to relieve pain; (6) patient satisfaction, apprehension, and complications associated with the perioperative period; and (7) type of department and hospital. The entire form is shown in Additional file 1. The medical students helped patients to complete the questionnaire form if needed.

Patients over 18 years of age, who were able to sign the consent form and fill out the questionnaire and VAS scale form, were included in the study. Furthermore, only participants who underwent major surgical procedures that required hospitalization were asked to participate.

The following patients were excluded from the study: (1) minors under 18 years of age, (2) patients admitted to the ICU (intensive care unit) after surgery, (3) patients who did not sign the written consent, (4) patients who were unable to fill out the form or mark pain severity on the VAS scale, (5) patients who received local anesthesia, and (6) patients who were not able to fill out the questionnaire form or the VAS form within 4 h after surgery.

The main variable measured in the study was the VAS value (given in mm) obtained at 4, 8, 12, and 24 h following the end of the surgery. We also analyzed the relationship between the pain intensity scores and the following parameters obtained during data collection: patient demographics, type of surgery, type of anesthesia, hospital type, kind of surgical ward, and operated region.

### Statistics

The analysis of variances (ANOVA), t-test, and multivariate regression were used for statistical analysis. Tukey’s HSD (honest significant difference) was used for post-hoc analysis. VAS results were presented as means and confidence intervals (CI). All measurements were performed using Statistica 12.5 software (StatSoft Inc., Tulsa, OK, USA).

## Results

### Hospitals and patients

The study was conducted from November 2015 to June 2016 in seven hospitals in eastern Poland, which were comprised of two university (284 patients), two secondary (154 patients), and three primary (local) (124 patients) hospitals. Each hospital was granted two weeks during the study period for data gathering. The VAS forms were collected from a total of 565 patients. Three patients were excluded from further analysis because of a local anesthesia procedure. The data regarding patient demographics and type of surgical ward are presented in Table 1.

**Table 1:** Patient demographics

	Number	Percent	Mean age	Height [cm]			Weight [kg]		
Women	269	47.8	52.78	162.42 (161.10–163.74)			71.82 (69.93–73.73)		
Men	293	52.2	50.78	174.10 (173.06–175.14)			82.68 (80.54–84.82)		
Overall	562	100	51.74	168.51 (167.55–169.47)			77.49 (75.98–78.99)		
Type of ward	Thoracic surgery	Gynecology	Orthopeady	Laryngology	Neurosurgery	Urology	Obstetrics	General surgery	Vascular surgery
Number of patients	113	82	112	75	22	42	16	70	25
Patients’ sex W/M (n)	35/78	82/0	50/62	23/52	10/12	13/29	16/0	33/37	4/21
Patients mean age	58.73	49.88	50.94	39.16	47.86	46.80	31.23	54.56	59.20

Patient demographics are presented as means and confidence intervals (CI).The second part of the table shows the number of patients (total and divided into sex) in every type of surgical department. *W* women, *M* men

### Total pain intensity

Pain severity is summarized in Table 2. The pain was described as moderate (40–59)) or severe (≥60).

**Table 2:** Patient pain intensity expressed by VAS at the 4, 8, 12 and 24 h after surgery

VAS	Mean (CI)	Moderate pain N (%)	Severe pain N (%)
4-h	34 (32–36)	221 (39.32)	111 (19.75)
8-h	32 (30–34)	187 (33.27)	81 (14.41)
12-h	27 (25–29)	135 (24.02)	67 (11.92)
24-h	25 (23–27)	116 (20.64)	48 (8.54)

*VAS* visual-analogue scale, *CI* confidence interval. Moderate and severe data are given as N and (%)

### Postoperative analgesics

The prevalence of analgesics used in the study is presented in Table 3. Approximately 91.3% (512/562) of patients received analgesics after surgery. Twenty-eight patients received strong opioids (morphine and nalbuphine), and tramadol was used in 150 cases (26.69%). Metamizole and ketoprofen were, by far, the most frequently administered analgesics for post-surgical pain. Of the patients, 8.7% (49/561) did not receive any drugs postoperatively.

**Table 3:** Analgesics used after surgery

Postoperative analgesics	number
no analgesics	49
acetylsalicylic acid	1
bupivacaine + fentanyl (epidural)	33
diclofenac	37
gabapentin	1
ibuprofen	1
ketoprofen	202
lidocaine	2
mefenamic acid	1
metamizol	384
morphine	27
nalbuphine	1
paracetamol	169
tramadol	150

Drugs are presented in alphabetical order

### Pain severity according to the type of hospital

**Fig. 1:**
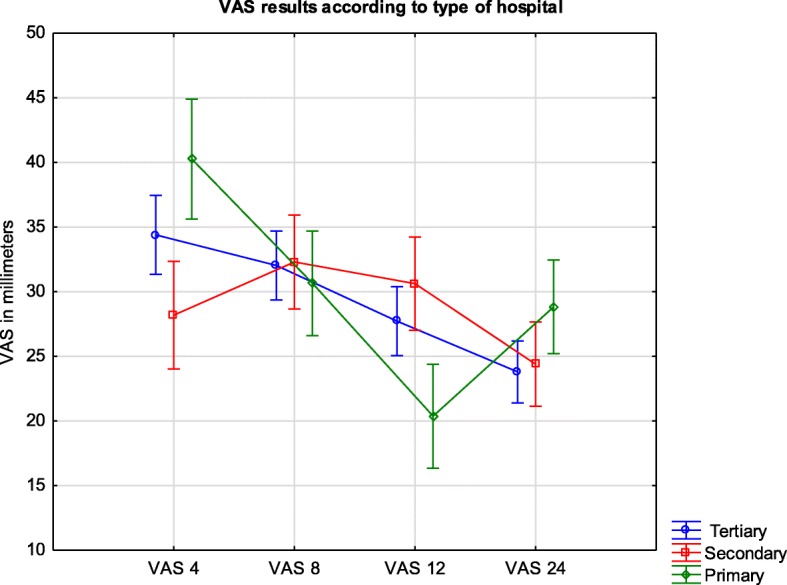
Legend: VAS results in millimeters during the first day after surgery in different types of hospitals. VAS are presented as means and confidence intervals

There was no difference in the overall pain intensity (measured with VAS) according to the reference type of hospital. However, significant difference was found between primary and secondary hospitals at the 4- and 12-h assessments (F = 10.77; *p* = 0.00001) (Fig. 1). The mean VAS at 4 h was 40 (36–45) for primary and 28 (24–32) for secondary hospitals (*p* = 0.0026). On the other hand, at 12 h after surgery, the mean VAS result was 20 (16–24) for primary and 31 (27–34) for secondary hospitals (*p* = 0.026).

### Pain severity according to the type of operated region

On data from the four VAS measurements, the highest level of pain was associated with surgery on the upper abdomen (36 (30–41)), while the lowest pain scores were found in patients undergoing surgical procedures in the head/neck region (26 (23–30) However, the difference was not significant (F = 2.09; *p* = 0.065).

At the 4-h measurement, the highest VAS results were obtained in patients undergoing thoracic (41 (37–46)) and upper abdomen procedures (43 (34–51)). These results were significantly higher than the lower limb VAS measurements (24 (18–29)) in our study (*p* = 0.00012 and 0.02).

A statistical difference was not found at the 8-h measurement, yet similar to previous assessments, the most painful procedures seemed to be associated with the upper abdomen (39 (31–46)) and, surprisingly, the lower limb (36 (31–41)).

At the 12-h measurement, there was a significant difference in VAS (*p* = 0.039) between surgeries of the chest (21 (17–25)) and lower limb (31 (27–36)).

**Fig. 2:**
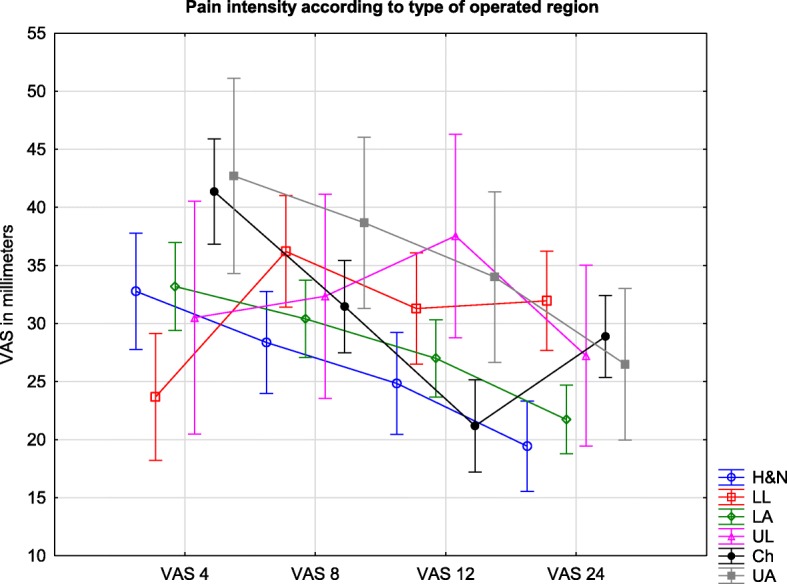
Legend: Pain intensity measured by VAS in millimeters during the first day after surgery according to operated region. VAS are presented as means and confidence intervals. H&N – head&neck; LL – lower limb; LA – lower abdomen; UL – upper limb; Ch – chest; upper abdomen

Furthermore, this tendency was maintained at the 24-h measurement, and lower limb procedures were associated with the highest pain intensity scores (32 (28–36)). A significant difference was demonstrated between lower limb surgeries and head/neck (19 (16–23) *p* = 0.00064) and hypogastrium operations (22 (19–25) *p* = 0.011). The results are presented in Fig. 2.

### Pain severity according to the type of surgical ward

The total difference in VAS values (mean of the four measurements) between patients treated in vascular surgery (19 (13–26)) and thoracic surgery (30 (27–33)) were significant (F = 2.49, *p* = 0.012).

**Fig. 3:**
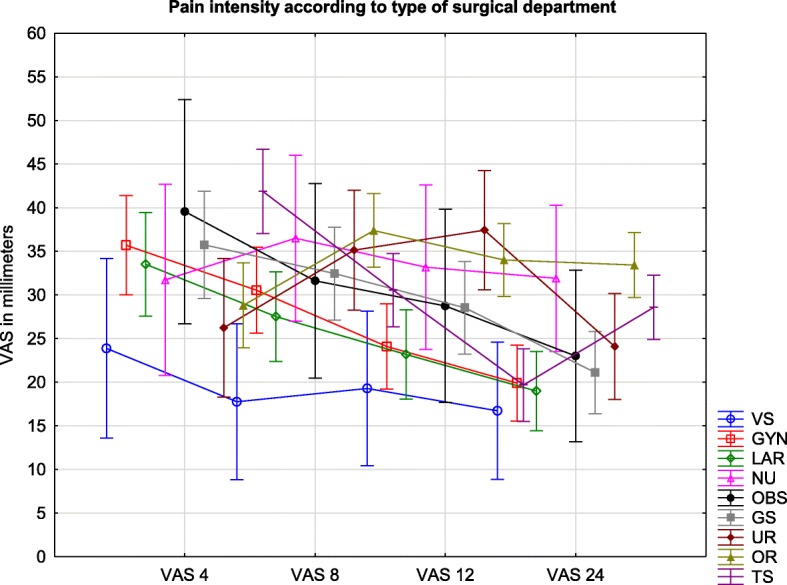
Legend: Pain intensity measured by VAS in millimeters during the first day after surgery according to surgical ward. VAS are presented as means and confidence intervals. VS – vascular surgery, GYN – gynecology; LAR – laryngology; NU – neurosurgery; OBS – obstetrics; GS – general surgery; UR – urology; OR – orthopedic surgery; TS – Thoracic surgery

At the 4-h measurement, a difference was found between orthopedic (29 (24–34)) and thoracic surgeries (42 (37–47); *p* = 0.0058). VAS results at the 8-h measurement demonstrated no statistical significance between studied wards. At the 12-h assessment, there was a significant difference in VAS scores between the thoracic (20 (16–24)) and the orthopedic (34 (30–38); *p* = 0.0001) and urology procedures (37 (31–44); *p* = 0.0093). Furthermore, at the 24-h VAS assessment, orthopedic procedures (33 (30–37)) seemed to be the most painful, especially in comparison to gynecology (20 (16–24); *p* = 0.0005), laryngology (19 (14–24); *p* = 0.00037)), and general surgery procedures (21 (16–26); *p* = 0.0085). The results are presented in Fig. 3.

### Type of anesthesia and pain severity

The types of anesthesia that were performed in our study are presented in Table 4.

**Table 4:** Number and percent of anesthesia types performed in the study

Type	Number (n)	Percent
General	335	59.61
Subarachnoid	111	19.75
Epidural	31	5.52
Peripheral regional	32	5.69
Combined spinal&epidural	4	0.71
General + epidural	8	1.42
General + peripheral regional	33	5.87
Subarachnoid + peripheral regional	8	1.42

**Fig. 4:**
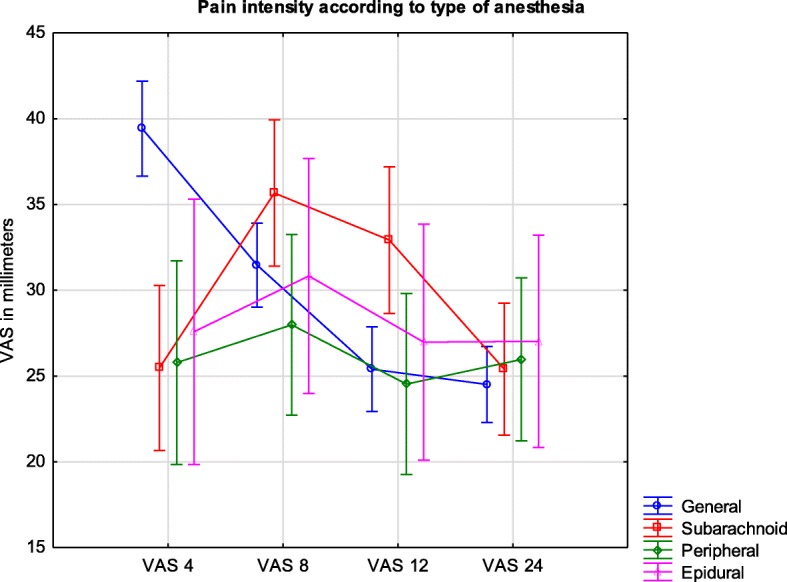
Legend: Pain intensity measured by VAS in millimeters during the first day after surgery in different types of anesthesia. VAS are presented as means and confidence intervals

For statistical analysis, in order to avoid bias associated with the small number of cases in some groups, patients were assigned to four groups as follows: general anesthesia (335), subarachnoid (111), epidural (43), and regional peripheral (73) (Fig. 4).

At the 4-h VAS measurement, a difference was found between the general anesthesia group (39 (37–42)) and the subarachnoid (26 (21–30); *p* = 0.00033) and peripheral regional groups (26 (20–32); *p* = 0.0077). No difference was found at the 8-h measurement; however, the most severe pain was noted in the subarachnoid group (36 (31–40)). Furthermore, statistical significance was detected at the 12-h VAS measurement between the subarachnoid (33 (29–37) and general anesthesia groups (24 (23–28); *p* = 0.017). No difference was found between groups at the 24-h VAS measurement.

### Pain severity according to the patient’s sex

**Fig. 5:**
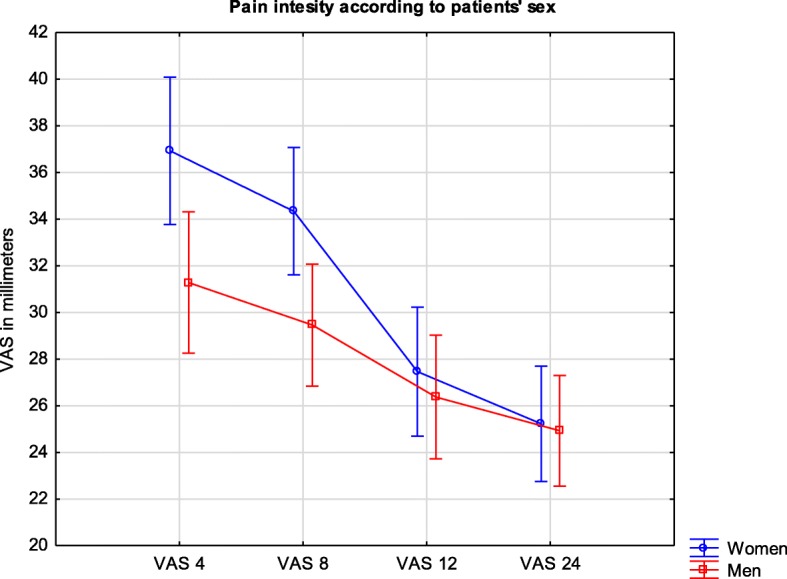
Legend: Pain intensity measured by VAS in millimeters during the first day after surgery according to sex. VAS are presented as means and confidence intervals

There was a significant difference in overall pain intensity according to the patient’s sex (Fig. 5). Women experienced more intense pain than men, with VAS values of 31 (29–33) and 28 (26–30), respectively; *p* = 0.044. However, that difference was only significant during the two first measurements (at 4 and 8 h). At the 4-h measurement, the mean VAS results for women and men subsequently reached 37 (34–40) and 31 (28–34), respectively; *p* = 0.012. At the 8-h measurement, the VAS values were 34 (32–37) for women and 30 (27–32) for men (p = 0.012).

### Correlation between patient’s age and VAS value

A statistical correlation was found at the 12-h measurement on the linear regression model between the patient’s age and VAS value. Although the *p*-value was 0.0027, the calculated r^2^ value was 0.0159. However, after dividing the participants of our study into three groups, younger (up to 45 years), middle-aged (45–60 years), and older patients (over 60 years), a significant difference was found between the older patients (21 (18–24), *n* = 180) and the younger (29 (26–33), *n* = 176; *p* = 0.0031) and middle-aged patients (30 (26–33), *n* = 205; *p* = 0.0022).

### Patient satisfaction with pain control

Of the 562 patients, 526 (93.6%) were satisfied with the quality of postoperative pain control (good or very good marks in the questionnaire). In contrast, 33 patients were not satisfied with the quality of postoperative pain control (poor marks), and five ranked the postoperative analgesia as not satisfactory. Overall VAS values (given as a mean of the four measurements) reached 51 (45–57) among the patients with poor pain control and was significantly higher than in the patients with satisfactory pain control (28 (27–30)).

### Impact of patient’s preoperative concerns on postoperative pain control

Patients who were afraid of poor postoperative pain control had slightly higher overall VAS values (32 (29–35)) in comparison to participants with no preoperative concerns (26 (22–30)), but these results were not significant.

However, when comparing VAS values at the specific time measurements, statistically significant differences were found. At the 4-h VAS measurement, there was a significant difference in pain intensity between patients who were afraid of either awareness or intraoperative pain (22 (13–32)) and those whose main concern was death (40 (34–45); *p* = 0.038) or postoperative pain (39 (34–45); *p* = 0.032). The same difference was observed at the 8-h measurement, but it was not significant. At the 12-h measurement, patients whose main concern was awareness indicated pain severity on the VAS score as the most intense (contrary to the previous observations), but this result was not significant. Furthermore, at the 24-h measurement, no difference in VAS scores was found, but slightly reduced pain was noted among the patients who were afraid of perioperative death (22.1 (17.8–26.3)).

## Discussion

Our results highlighted new aspects of postoperative pain control, such as unsatisfactory analgesia of patients with single-shot subarachnoid anesthesia at 8 and 12 h after the surgery and a low number of strong opioids administered in the hospitals studied.

The prevalence of moderate and severe pain was high among the study population, especially during the first hours after the surgery (39 for the 4-h VAS). However, at the same time, 93.6% of patients recognized pain control as good or very good.

Of the patients, 50.5% were anesthetized in two university hospitals. Lower numbers of surgeries in low-volume units might have been more vulnerable to random deviations during statistical analysis. However, the impact of the patient distribution in our study is difficult to predict. The difference in pain intensity between primary and secondary hospitals at 4- and 12- h after surgery became evident after further analysis and is likely due to the presence of orthopedic wards and the quantity of these surgeries performed in secondary hospitals. Furthermore, the probable cause of the 12-h peak in VAS values in orthopedic patients, with the high prevalence of lower limb operations, was the use of subarachnoid anesthesia. Although spinal, single-shot anesthesia is a standard approach for hip and knee surgeries, and more intense pain at the 12-h measurement was a result of insufficient supplementary analgesic techniques. Currently, the early mobilization of patients after hip or knee replacements is one of the main goals and possesses beneficial effects [11, 12].

The combined spinal-epidural technique or systemic opioid administration may postpone the rehabilitation process and delay the patient’s discharge. The femoral nerve, lumbar plexus, or adductor canal blocks are well-established alternatives [13, 14]. However, in our study, such analgesic techniques were only implemented in 11 cases.

Younger age of patients seems to be an independent risk factor for higher postoperative pain in our investigation. Nevertheless, such a difference was not presented in the study of Gagliese and Katz if non-verbal scales, such as VAS or NRS were utilized [15]. Conversely, in the study of Sommer et al., as well as two more recent studies from Germany, a difference was found between younger and older patients (younger perceived more intense pain) [10, 16, 17].

Moreover, the authors of these articles identified women as more vulnerable to postoperative pain. These results are consistent with those found by our study.

Although opioid-related side effects are common, omitting these drugs in the postoperative period can lead to augmentation of pain. In the very recent study of Thiels et al., 93.9% of surgical patients received opioid prescriptions at discharge [18]. In contrast, only 4.9% of participants in our study obtained strong opioids (morphine and nalbuphine) during the first 24 h after surgery. Even though the results presented by Breivik et al. regarding chronic pain in Europe showed that Polish patients used the lowest number of opioids and the highest number of non-opioid drugs in comparison to other European patients, the low amount of parenteral opioids used in our study was unexpected [19].

The Polish national guidelines for post-operative pain management present multiple analgesic methods and regimens [6]. The guidelines emphasize the role of multimodal analgesia, as well as the broader implementation of loco-regional techniques. This statement is consistent with recommendations of the American Pain Society [20]. The other aspect presented in the Polish guidelines is preemptive analgesia and regular administration of painkillers, not only on the patient’s demand. However, there is a discrepancy between our results and the guidelines. The majority of our patients did not receive preemptive or multimodal analgesia. Moreover, in many cases, drugs were not administered at regular intervals. Only 13% of our study participants received peripheral regional anesthesia/analgesia methods. We suppose that the main reason for this fact is insufficient time spent with patients due to the lack of staff in most hospitals. Moreover, drugs are not administered according to VAS or NRS results, but by medical staff habits [9]. The improvement of communication and cooperation between nurses and physicians could be the solution in many clinical scenarios, and the majority of problems could be solved by the organization of specialized teams for acute pain management and continuous training of healthcare providers.

## Conclusions

Results presented in our study show that many patients still experience moderate or severe pain in the postoperative period, even though there are guidelines and methods to treat pain after surgical procedures. Moreover, postoperative pain control seems to be unexpectedly poor after single-shot subarachnoid anesthesia. Despite concerns, the use of analgesics may be insufficient in some groups of patients. Our study also indicates new variables that influence the severity of pain, such as operated region, anesthetic technique, and the type of surgical department. The results obtained in our study are in discrepancy with recommendations presented by the national guidelines for post-operative pain management.

## Additional file

Additional file 1: Questionnaire form. (DOCX 15 kb)

## Abbreviations

ANOVA: The analysis of variances
CI: confidence interval
N: number
NRS: numerical rating scale
VAS: visual-analogue scale

## References

[1] Weiser TG, Regenbogen SE, Thompson KD (2008). An estimation of the global volume of surgery: a modelling strategy based on available data. Lancet.

[2] Brennan F, Carr DB, Cousins M (2007). Pain management: a fundamental human right. Anesth Analg.

[3] Apfelbaum JL, Chen C, Mehta SS, Gan TJ (2003). Postoperative pain experience: results from a national survey suggestpostoperative pain continues to be undermanaged. AnesthAnalg.

[4] Sauaia A, Min SJ, Leber C, Erbacher K, Abrams F, Fink R (2005). Postoperative pain management in elderly patients: correlation between adherence to treatment guidelines and patient satisfaction. J Am Geriatr Soc.

[5] Gan T, Habib A, Miller T, White W, Apfelbaum J (2014). Incidence, patientsatisfaction, and perceptions of post-surgical pain: results from a US nationalsurvey. Curr Med Res Opin.

[6] Misiołek H, Cettler M, Woroń J (2014). The 2014 guidelines for post-operative pain management. Anaesthesiol Intensive Ther.

[7] Erlenwein J, Stamer U, Koschwitz R (2014). Inpatient acute pain management in German hospitals: results from the national survey “Akutschmerzzensus 2012”. Schmerz.

[8] Tomaszek L, Dębska G. Knowledge, compliance with good clinical practices and barriers to effective control of postoperative pain among nurses from hospitals with and without a "hospital without pain" certificate. J Clin Nurs. 2017 Dec 8; 10.1111/jocn.14215. 10.1111/jocn.14215 29218814

[9] Mędrzycka-Dąbrowska WA, Dąbrowski S, Basiński A, Pilch D (2016). Perception of barriers to postoperative pain management in elderly patients in polish hospitals with and without a “hospital without pain” certificate – a multi-center study. Archives of Medical Science : AMS.

[10] Meißner W, Komann M, Erlenwein J (2017). The quality of postoperative pain therapy in German hospitals. —the effect of structural and procedural variables Dtsch Arztebl Int.

[11] Chandrasekaran S, Ariaretnam SK, Tsung J (2009). Early mobilization after total knee replacement reduces the incidence of deep venous thrombosis. ANZ J Surg.

[12] Guerra ML, Singh PJ, Taylor NF (2015). Early mobilization of patients who have had a hip or knee joint replacement reduces length of stay in hospital: a systematic review. Clin Rehabil.

[13] Chan EY, Fransen M, Parker DA (2014). Femoral nerve blocks for acute postoperative pain after knee replacement surgery. Cochrane Database Syst Rev.

[14] Amiri HR, Zamani MM, Safari S. Lumbar plexus block for Management of hip Surgeries. Anesth Pain Med2014;4(3):e19407. 10.5812/aapm.19407 PMC4183079 25289374

[15] Gagliese L, Katz J (2003). Age differences in postoperative pain are scale dependent: a comparison of measures of pain intensity and quality in younger and older surgical patients. Pain.

[16] Sommer M, de Rijke JM, van Kleef M (2008). The prevalence of postoperative pain in a sample of 1490 surgical inpatients. EurJ Anaesthesiol.

[17] Gerbershagen HJ, Pogatzki-Zahn E, Aduckathil S (2014). Procedure specific risk factor analysis for the development of severe postoperative pain. Anesthesiology.

[18] Thiels CA, Anderson SS, Ubl DS (2017). Wide variation and over prescription of opioids after elective surgery. Ann Surg.

[19] Breivik H, Collett B, Ventafridda V (2006). Survey of chronic pain in Europe: prevalence, impact on daily life, and treatment. EurJ Pain.

[20] Chou R, Gordon DB, de Leon-Casasola OA, Rosenberg JM, Bickler S (2016). Management of Postoperative Pain: a clinical practice guideline from the American pain society, the American Society of Regional Anesthesia and Pain Medicine, and the American Society of Anesthesiologists' committee on regional anesthesia, executive committee, and administrative council. J Pain.

