# Supplementary material for: Survey of postoperative pain control in different types of hospitals: a multicenter observational study
Source: BMC Anesthesiol. 2018 Jul 18;18:83. doi: 10.1186/s12871-018-0551-3 (PMC6052639; doi:10.1186/s12871-018-0551-3)
Supplement: Supplementary file 1 — Questionnaire form. (DOCX 15 kb) [file 12871_2018_551_MOESM1_ESM.docx]

Additional file 1: Questionnaire form

1. patient’s demographics
   1. Sex: F/M
   2. Age: Weight:
   3. Height:
2. Operated region
   1. Head and neck
   2. Chest
   3. Epigastrium
   4. Hypogastrium
   5. Upper limb
   6. Lower limb
3. Type of anesthesia
   1. General
   2. Regional
      1. Subarachnoid
      2. Epidural
      3. Plexuses or peripheral nerves
4. Pain severity with VAS in millimeters
   1. 0-4 h
   2. 4-8 h
   3. 8-12 h
   4. 24 h
5. Drugs and pain-relief techniques used during the first day
   1. Nonopioid drugs
   2. Opioids
   3. Routs of administration
   4. Regional analgesia (including continuous techniques)
   5. Patient-controlled analgesia (PCA)
6. Patients’ satisfaction and apprehension
   1. How do you asses the quality of pain treatment?
      1. Very good, no pain at all
      2. Good, pain was much lower after treatment
      3. Satisfactory, pain was slightly reduced after treatment
      4. Unsatisfactory, pain was not lower after treatment
   2. Before the surgery my highest concern was:
      1. The effectiveness of operation
      2. Awareness and pain during operation
      3. Pain after procedure
      4. Death or not being awakened after operation
   3. During the first day after the surgery my problems included (may mark more than one option)
      1. Nausea
      2. Vomiting
      3. Urinary catheter
      4. Breathlessness
      5. Dizziness
      6. Constipation
      7. Itching
      8. Sleeplessness
      9. Other:
7. Type of hospital and department:
   1. Hospital: primary, secondary, tertiary
   2. Department:
